# A positive feedback loop of lncRNA-RMRP/ZNRF3 axis and Wnt/β-catenin signaling regulates the progression and temozolomide resistance in glioma

**DOI:** 10.1038/s41419-021-04245-y

**Published:** 2021-10-16

**Authors:** Tie Liu, Jie Hu, Bo Han, Shishan Tan, Wenqing Jia, Yu Xin

**Affiliations:** Department of Neurosurgery, Beijing Tiantan Hospital, Capital Medical University, Beijing, 100070 China

**Keywords:** Drug regulation, Long non-coding RNAs

## Abstract

Drug resistance strikingly limits the therapeutic effect of temozolomide (TMZ) (a common drug for glioma). Long non-coding RNA (lncRNA) RMRP has been found to be implicated in glioma progression. However, the effect of RMRP on TMZ resistance along with related molecular mechanisms is poorly defined in glioma. In the present study, RMRP, ZNRF3, and IGF2BP3 were screened out by bioinformatics analysis. The expression levels of lncRNAs and mRNAs were measured by RT-qPCR assay. Protein levels of genes were detected by western blot and immunofluorescence assays. ZNRF3 mRNA stability was analyzed using Actinomycin D assay. Cell proliferative ability and survival rate were determined by CCK-8 assay. Cell apoptotic pattern was estimated by flow cytometry. The effect of RMRP knockdown on the growth of TMZ-treated glioma xenograft tumors was explored in vivo. The relationships of IGF2BP3, RMRP, and ZNRF3 were explored by bioinformatics prediction analysis, RNA immunoprecipitation, luciferase, and RNA pull-down, and chromatin immunoprecipitation assays. The results showed that RMRP was highly expressed in glioma. RMRP knockdown curbed cell proliferation, facilitated cell apoptosis and reduced TMZ resistance in glioma cells, and hindered the growth of TMZ-treated glioma xenograft tumors. RMRP exerted its functions by down-regulating ZNRF3 in glioma cells. IGF2BP3 interacted with RMRP and ZNRF3 mRNA. IGF2BP3 knockdown weakened the interaction of Argonaute 2 (Ago2) and ZNRF3. RMRP reduced ZNRF3 expression and mRNA stability by IGF2BP3. RMRP knockdown inhibited β-catenin expression by up-regulating ZNRF3. The inhibition of Wnt/β-catenin signaling pathway by XAV-939 weakened RMRP-mediated TMZ resistance in glioma cells. β-catenin promoted RMRP expression by TCF4 in glioma cells. In conclusion, RMRP/ZNRF3 axis and Wnt/β-catenin signaling formed a positive feedback loop to regulate TMZ resistance in glioma. The sustained activation of Wnt/β-catenin signaling by RMRP might contribute to the better management of cancers.

## Introduction

Glioma is the commonest brain and central nervous system malignant tumor, accounting for approximately 80% of all malignant intracranial tumor cases [1]. More than 50% of glioma cases are diagnosed at the late stage (WHO grade IV glioblastoma), while glioblastoma patients after routine treatment have a poor prognosis with a 5-year survival rate of less than 5% [1, 2]. In addition, the recurrence rate of glioma is very high, and most low-grade glioma will recur post initial treatment and transform into high-grade glioma [3–5].

Chemotherapy is a common clinical therapeutic strategy for glioma [6, 7]. Temozolomide (TMZ), an orally bioavailable DNA alkylating agent, has been a backbone of glioma treatment for more than 20 years due to its ability to penetrate the blood-brain barrier [8, 9]. TMZ exerts its anti-tumor activity mainly by inducing base mismatches, DNA repair aberration, DNA strand breaks, and cell death [9]. However, TMZ only can slightly increase the survival of patients with glioblastoma and many patients with glioblastoma have poor or no responses to TMZ, which is mainly caused by inherent and acquired TMZ resistance [10, 11]. Thus, it is imperative to clarify the molecular mechanisms of TMZ resistance and identify novel targets to improve its therapeutic efficacy.

Long non-coding RNAs (lncRNAs) are a group of RNA transcripts longer than 200 nucleotides (nt) in length that lack protein-coding potential [12, 13]. LncRNAs have been found to be involved in the tumor onset and progression of multiple malignancies including glioma [13, 14]. Moreover, dysregulated lncRNAs in clinical glioma samples are closely associated with tumor grade, tumor malignant phenotypes, and patients’ survival, which have potential values in the diagnosis, treatment, and prognosis of glioma [14–16]. LncRNAs can exert their functions through regulating almost all aspects of gene expression such as RNA processing, mRNA stability/modification/translation [15, 17].

LncRNA RNA component of mitochondrial RNA processing endoribonuclease (RMRP) has been reported to be an oncogenic factor in multiple cancers such as multiple myeloma [18], neonatal neuroblastoma [19], and non-small-cell lung cancer [20]. Also, a recent study demonstrated that RMRP was highly expressed in glioma tissues and RMRP knockdown led to the marked reduction of cell proliferative, migratory and invasive abilities and a notable increase of cell apoptotic rate in glioma [21]. However, it remains unknown whether RMRP is involved in the regulation of TMZ resistance in glioma. In addition, the molecular mechanisms of RMRP underlying TMZ resistance have not been examined.

In this text, we investigated the effects of RMRP knockdown on glioma cell proliferation, apoptosis, and TMZ resistance in vitro and the growth of TMZ-treated glioma xenograft tumors in vivo. Also, downstream targets and related regulators of RMRP were further explored.

## Materials and methods

### Clinical samples

Glioma tissues were obtained by surgical excision from glioma patients without any treatment (*n* = 20, 20–50 years old) and age-matched patients with relapsed glioma after TMZ-based chemotherapy (*n* = 12, 20–50 years old) from July 2018 to December 2018 at Beijing Tiantan Hospital of Capital Medical University. These patients enrolled in our project only suffered from glioma. Inclusive criteria: (1) KPS score >60, (2) no obvious abnormality in a routine blood test, (3) no noticeable impairment in organ (e.g. liver, kidney, and heart) functions, and (4) no serious postoperative complication. Glioma patients with other diseases and poor general health conditions and patients during pregnancy were excluded from our project. Our study was approved by the Medical Ethics Committee of Beijing Tiantan Hospital of Capital Medical University (No. KYSQ 2020-271-01). Also, written informed consents were obtained from all patients before experiments.

### Reagents

Small interference RNAs (siRNAs) targeting RMRP, ZNRF3, and IGF2BP3 and a scrambled control siRNA (si-NC) were synthesized by GenePharma Co., Ltd. (Shanghai, China). Sense siRNA sequences were provided in Supplementary Table 1. Overexpression plasmids were purchased from Sangon Biotech Co., Ltd. (Shanghai, China). TMZ was purchased from MedChemExpress Co., Ltd. (Monmouth Junction, NJ, USA).

### Cell culture and transfection

U251 cells were purchased from the Cell Bank of the Chinese Academy of Sciences (Shanghai, China) and cultured in Dulbecco’s Modified Eagle’s Medium (DMEM) (Thermo Fisher Scientific, Rockford, IL, USA) supplemented with 10% fetal bovine serum (FBS, Thermo Fisher Scientific). LN229 cells were obtained from American Type Culture Collection (ATCC, Manassas, VA, USA) and grown in DMEM medium (Thermo Fisher Scientific) containing 5% FBS (Thermo Fisher Scientific). TMZ-resistant U251 cell line (U251/TMZ) and TMZ-resistant LN229 cell line (LN229/TMZ) were established by continuous exposure to increasing doses of TMZ. All cells were maintained in a 95% air/5% CO_2_ incubator at 37 °C. siRNAs or plasmids, alone or in combination, were transfected into U251 and LN229 cells using Lipofectamine 3000 reagent (Thermo Fisher Scientific) following the protocols of the manufacturer.

### RT-qPCR assay

Total RNA was extracted from glioma tissues and cells using Trizol reagent (Thermo Fisher Scientific) following the instructions of the manufacturer. Next, the first-strand cDNA was synthesized using SuperScriptIII Reverse Transcriptase (Thermo Fisher Scientific), random primers, and RNA template. Subsequently, quantitative PCR reactions were performed using SYBR Green PCR Master Mix (Thermo Fisher Scientific), cDNA template, and specific PCR primers for RMRP, HOXA-AS3, CASC9, ZNRF3, and GAPDH. GAPDH acted as the housekeeping gene to normalize the expression of other genes. The quantitative PCR primer sequences were presented in Supplementary Table 2.

### CCK-8 assay

Cell proliferative ability and survival rate were measured using a CCK-8 kit (Beyotime Biotechnology, Shanghai, China). For the detection of cell proliferative activity, transfected cell suspensions (100 µl/well) were seeded into 96-well plates and then co-incubated with CCK-8 solution (10 µl/well) at 0, 24, 48, 72 h after plating. After 3 h of co-incubation, the optical density (OD) values were measured at 450 nm using the SpectraMax 190 microplate reader (Molecular Device, San Jose, CA, USA). For the measurement of cell survival rate, transfected cells (100 µl/well) were seeded into 96-well plates for 24 h and then treated with different concentrations (8, 16, 32, 64, 128, or 256 µM) of TMZ for another 24 h. Next, 10 µl of CCK-8 solution was added to each well. Three hours later, OD values were determined at 450 nm, followed by the determination of 50% inhibitory concentration (IC_50_). Cell survival rate (%) = (OD (drug-treated group)-OD (blank group))/(OD (untreated group)-OD (blank group))×100%.

### Cell apoptotic rate detection

Cell apoptotic rate was estimated using the Annexin V-FITC Apoptosis Detection Kit (Beyotime Biotechnology) according to the manufacturer’s instructions. Briefly, cells were collected at 48 h after transfection and re-suspended in Annexin V-FITC binding solution. Next, cells were co-incubated with Annexin V-FITC and Propidium Iodide (PI) solutions for 15 min at room temperature in a dark place. Next, stained samples were placed in an ice bath and cell apoptotic proportion was determined using flow cytometry (BD Biosciences, San Diego, CA, USA).

### In vivo experiments

Lentiviruses carrying interference fragment targeting RMRP (sh-RMRP) and lentiviruses bearing scramble sequences (sh-NC) were obtained from Novobio Biotech Co., Ltd. (Shanghai, China). U251/TMZ cells were infected with RMRP-targeting or non-targeting lentiviruses for 3 days and then screened with puromycin (1 μg/ml) for 1 week to establish cell lines with or without RMRP stable knockdown.

Mouse experiments were conducted with the approval of the Animal Care and Use Committee of Beijing Tiantan Hospital of Capital Medical University and performed with the standard experimental procedures. The BALB/C nude mice (male, 6-weeks-old) were obtained from the Laboratory Animal Center of Beijing Tiantan Hospital of Capital Medical University and acclimatized to the new surroundings for 1 week. Mice with poor mental and physical health status were excluded from our project. Mice were randomly divided into control, sh-NC, and sh-RMRP groups by a researcher who was blinded to our experimental design. Each group contains 6 mice. U251/TMZ cells (1 × 10^7^ cells/mouse), U251/TMZ cells infected with sh-NC (1 × 10^7^ cells/mouse), or U251/TMZ cells infected with sh-RMRP (1 × 10^7^ cells/mouse) were injected subcutaneously into the right-back of mice in the control, sh-NC, or sh-RMRP group, respectively. TMZ (25 mg/kg body weight) was intraperitoneally injected into mice every other day for 14 days when tumor volumes reached approximately 100 mm^3^. Tumor volume was monitored using a vernier caliper every 2 days and calculated using the following formula: tumor volume (mm^3^) = (L × W^2^)/2, where L and W respectively represent the tumor’s length and width. Tumors were resected, photographed, and weighed at the end of the experiments.

### Subcellular localization analysis of RMRP

Cytoplasmic and nuclear RNA was isolated from U251 and LN229 cells using Cytoplasmic & Nuclear RNA Purification Kit (Norgen Biotek Inc., Thorold, Canada) following the protocols of the manufacturer. Next, the levels of GAPDH, RMRP, and U6 snRNA in cytoplasm and nucleus were determined through RT-qPCR assay.

### Western blot analysis

U251 and LN229 cells were lysed using RIPA lysis buffer (Solarbio Life Sciences, Beijing, China) containing protease inhibitor cocktail (Sigma-Aldrich, St Louis, MO, USA). After high-speed centrifugation (12000 rpm, 15 min, 4 °C), cell supernatants were collected. The concentration of protein in cell supernatants was measured using a Bio-Rad Bradford Protein Assay Kit (Bio-Rad Laboratories, Hercules, CA, USA). Next, protein (30 μg/sample) was separated by sodium dodecyl sulfate-polyacrylamide gel electrophoresis and transferred on nitrocellulose membranes (Millipore, Bedford, MA, USA). The membranes were sequentially incubated with 5% non-fat milk for 1 h at room temperature, the primary antibody against ZNRF3 (cat. no. orb386705, Biorbyt, Cambridge, UK), IGF2BP3 (cat. no. 57145 S, Cell Signaling Technology, Danvers, MA, US), β-catenin (cat. no. 9582 S, Cell Signaling Technology), GAPDH (cat. no. 5174 T, Cell Signaling Technology), or Tubulin (cat. no. 5335 S, Cell Signaling Technology) for 12 h at 4 °C, and the corresponding horseradish peroxidase-conjugated secondary antibody for 1 h at room temperature. Next, the membranes were exposed to the Pierce ECL Western Blotting Substrate (Thermo Scientific) to detect the protein signals.

### mRNA stability analysis

ZNRF3 mRNA stability was measured using actinomycin D (ActD, Sigma-Aldrich, St. Louis, MO, USA) assay. Briefly, 5 µg/ml of ActD was added into the cell medium at 24 h post-transfection. At 0, 2, 4, 6 h after ActD treatment, RNA was isolated from cells and ZNRF3 mRNA level was tested through RT-qPCR assay.

### RNA immunoprecipitation (RIP) assay

RIP assay was carried out in U251 cells using the Magna RIP RNA-Binding Protein Immunoprecipitation Kit (Millipore, Temecula, CA, USA) and antibody against IGF2BP3 or IgG following the manufacturer’s protocols. RMRP and ZNRF3 mRNA levels enriched by IGF2BP3 or IgG antibody were measured through RT-qPCR assay.

### RNA pull-down assay

RNA pull-down assay was performed using a Pierce Magnetic RNA-Protein Pull-Down Kit (Thermo Fisher Scientific) according to the manufacturer’s protocols. Biotinylated ZNRF3 3’UTR fragment 1 (1–2477 nt), fragment 2 (2445-3539 nt), and fragment 3 (3506–3912 nt) were purchased from Wuhan Genecreate CO., Ltd. (Wuhan, China). Briefly, biotin-labeled RNAs were captured by streptavidin-conjugated magnetic beads. Next, the RNA-bead complexes were co-incubated overnight at 4 °C with the lysates of U251 cells. Finally, RNA-conjugated proteins were eluted and the protein level of IGF2BP3 was determined by western blot assay.

### Immunofluorescence (IF) assay

Cells were fixed with 4% paraformaldehyde solution for 15 min and then treated with 0.5% Triton X-100 solution for 20 min. Next, samples on the slides were blocked with normal goat serum (BOSTER Biological Technology Co., Ltd., Wuhan, China) for 30 min at room temperature and then incubated overnight with primary antibody against β-catenin at 4 °C. On the next day, samples were incubated with FITC-labeled goat anti-rabbit IgG (Proteintech Group, Inc., Wuhan, China) for 1 h at 37 °C in the dark. Next, cell nuclei were stained with DAPI solution (Beyotime Biotechnology) for 5 min under dark conditions. After being mounted with Fluoromount-G (Birmingham, AL, USA), the slides were imaged using the Olympus BX53 microscope (Olympus Optical Co. Ltd., Tokyo, Japan).

### Luciferase reporter assay

TCF/LEF1-Luc reporter was purchased from Genomeditech Co. Ltd. (Shanghai, China). A partial fragment of RMRP gene promoter containing putative TCF4 binding site 1, 2, or 3 was constructed into a pGL3-Luc reporter by Genecreate Co., Ltd. (Wuhan, China) and generated recombinant plasmid was named pGL3-luc-TBE1, pGL3-luc-TBE2, or pGL3-luc-TBE3, respectively. Also, pGL3-luc-TBE3 reporter (MUT) with mutant TCF4 binding site 3 was constructed by Genecreate Co., Ltd. LN-229 cells were co-transfected with a recombinant reporter, pRL-TK Renilla luciferase plasmid and pcDNA3.1-TCF4 overexpression plasmid/pcDNA3.1-empty vector. Next, a Dual-Luciferase Reporter Assay (Promega, Madison, WI, USA) kit was used to measure the luciferase activities at 48-h post-transfection with Renilla luciferase activity as the internal control.

### Chromatin immunoprecipitation (ChIP) assay

CHIP assay was performed using the CHIP kit (Millipore) and TCF4 or IgG antibody (Proteintech Group) following the instructions of the manufacturer.

### Statistical analysis

Data were analyzed using GraphPad Prism 7 software (GraphPad Software Inc., La Jolla, CA, USA) with results presenting as means ± standard deviation. Differential analysis between groups was performed using a *t*-test. Differences among groups were compared using one-way ANOVA or two-way ANOVA along with the Bonferroni post hoc test. The variance is similar between the groups that are being statistically compared. Differences were regarded as statistically significant when the *P*-value was less than 0.05.

## Results

### Differential expression analysis of lncRNAs in glioma versus normal tissues and identification of interested lncRNAs

**Fig. 1:**
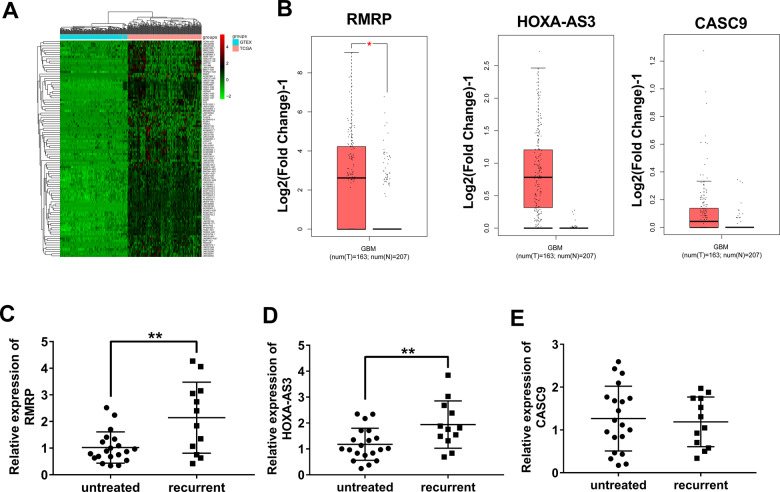
Expression profile analysis of lncRNAs in glioma and screening of interested lncRNAs. **A** The top 100 up-regulated lncRNAs in glioma versus normal brain tissues. **B** Expression analyses of 3 lncRNAs (RMRP, HOXA-AS3, and CASC9) in glioma versus normal tissues in the GEPIA database. **C** Expression levels of RMRP, HOXA-AS3, CASC9 in tumor tissues isolated from patients with recurrent glioma (*n* = 12) and glioma patients without any treatment (*n* = 20) were measured through RT-qPCR assay. *P < 0.05. **P < 0.01.

In this project, the expression data of lncRNAs in glioma tumor tissues (*n* = 168) and normal brain tissues (*n* = 150) were downloaded from TCGA and GTEx databases, respectively. Next, differentially expressed lncRNAs (|log_2_FoldChange | ≥1 and *P* < 0.05) were identified in glioma samples versus normal brain tissue samples. The top 100 up-regulated lncRNAs in glioma tissues were presented in Fig. 1A. After literature retrieval, 3 lncRNAs (RMRP, HOXA-AS3, and CASC9) related to glioma tumorigenesis and progression were screened out for further investigations. Moreover, GEPIA database analyses revealed that expression levels of RMRP, HOXA-AS3, and CASC9 were notably up-regulated in glioma tissues than in normal tissues (Fig. 1B). Given the association of tumor recurrence and drug resistance, expression patterns of RMRP, HOXA-AS3, and CASC9 were examined in tumor tissues isolated from glioma patients without any treatment (*n* = 20) and glioma patients suffered from recurrence post-treatment (*n* = 12). Results showed that RMRP and HOXA-AS3 expression levels were higher in tumors isolated from patients with relapsed glioma compared to the untreated group (Fig. 1C–E). To our knowledge, the association of HOXA-AS3 and drug resistance has been reported in two previous articles [22, 23]. which showed that HOXA-AS3 knockdown weakened the resistance of non-small-cell lung cancer and bladder cancer cells to cisplatin. However, no or few studies were performed to explore the correlation of RMRP and drug resistance in glioma. TMZ is a widely used chemotherapeutic drug for glioma patients [8, 9]. Hence, the roles and molecular basis of RMRP in glioma tumorigenesis and TMZ resistance were further investigated in the subsequent experiments.

### Effects of RMRP knockdown on glioma cell proliferation, apoptosis, and TMZ resistance

**Fig. 2:**
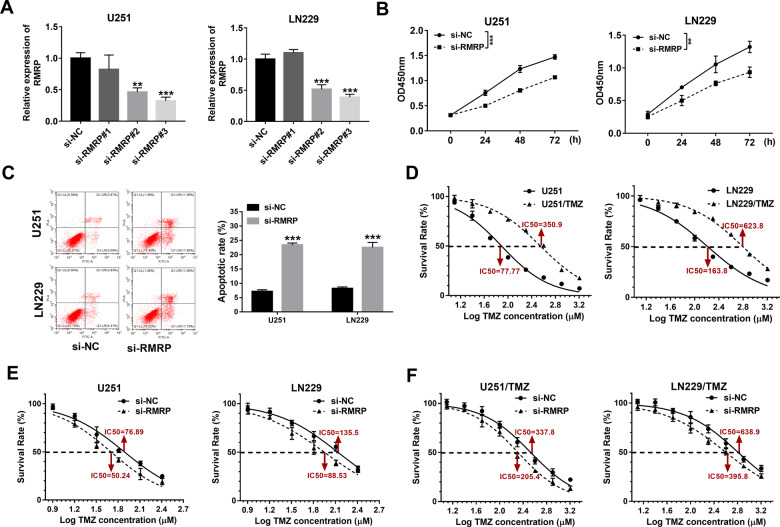
RMRP knockdown curbed cell proliferation, facilitated cell apoptosis, and reduced TMZ resistance in glioma cells. **A** U251 and LN229 cells were transfected with si-NC, si-RMRP#1, si-RMRP#2, or si-RMRP#3. Forty-eight hours later, RMRP level was examined through RT-qPCR assay (*n* = 3). **B** Cell proliferative ability was estimated through CCK-8 assay in U251 and LN229 cells transfected with si-NC or si-RMRP#3 at 0, 24, 48, 72 h post-plating (*n* = 3). **C** Cell apoptotic rate was determined by flow cytometry in U251 and LN229 cells transfected with si-NC or si-RMRP#3 at 48 h after transfection (*n* = 3). **D** U251, LN229, U251/TMZ, and LN229/TMZ cells were treated with different doses of TMZ for 24 h, followed by the measurement of TMZ IC_50_ values via CCK-8 assay (*n* = 3). **E** and **F** U251, LN229, U251/TMZ, and LN229/TMZ cells were transfected with si-NC or si-RMRP#3. Transfected cells were seeded into 96-well plates for 24 h and then treated with different concentrations of TMZ for an additional 24 h. Next, the cell survival rate was determined through the CCK-8 assay (*n* = 3). *P < 0.05. **P < 0.01. ***P < 0.001.

To have a deep insight into the functions of RMRP in glioma progression and TMZ resistance, 3 siRNAs targeting RMRP (si-RMRP#1, si-RMRP#2, and si-RMRP#3) and a scrambled control siRNA (si-NC) were synthesized. Subsequent transfection efficiency analyses revealed that the transfection of si-RMRP#2 or si-RMRP#3 could markedly reduce RMRP abundance in U251 and LN229 cells compared to the si-NC-transfected group (Fig. 2A). Considering the best knockdown effect of si-RMRP#3 on RMRP, si-RMRP#3 was selected for the following loss-of-function experiments. CCK-8 assay revealed that RMRP depletion strikingly weakened the proliferative ability of U251 and LN229 cells (Fig. 2B). Moreover, RMRP knockdown led to an approximately 2.7-fold increase in cell apoptotic percentage (Q1-UR + Q1-LR) in U251 cells (Fig. 2C). Also, an approximately 2.6-fold increase in cell apoptotic rate was observed in si-RMRP-transfected LN229 cells than that in the si-NC-transfected cells (Fig. 2C). In addition, TMZ-resistant glioma cell lines (U251/TMZ and LN229/TMZ) were successfully constructed, as evidenced by the marked increase of TMZ IC_50_ values in U251/TMZ and LN229/TMZ cells compared to corresponding parental cells (Fig. 2D). Furthermore, our data revealed that RMRP depletion led to the notable reduction of TMZ IC_50_ values in U251, LN229, U251/TMZ, and LN229/TMZ cells (Fig. 2E and F), suggesting that RMRP loss weakened TMZ resistance in parental and TMZ-resistant glioma cells. In summary, these outcomes presented that RMRP knockdown curbed cell proliferation, facilitated cell apoptosis, and reduced TMZ resistance in glioma cells.

### Effects of RMRP knockdown on the growth of TMZ-treated glioma xenograft tumors

**Fig. 3:**
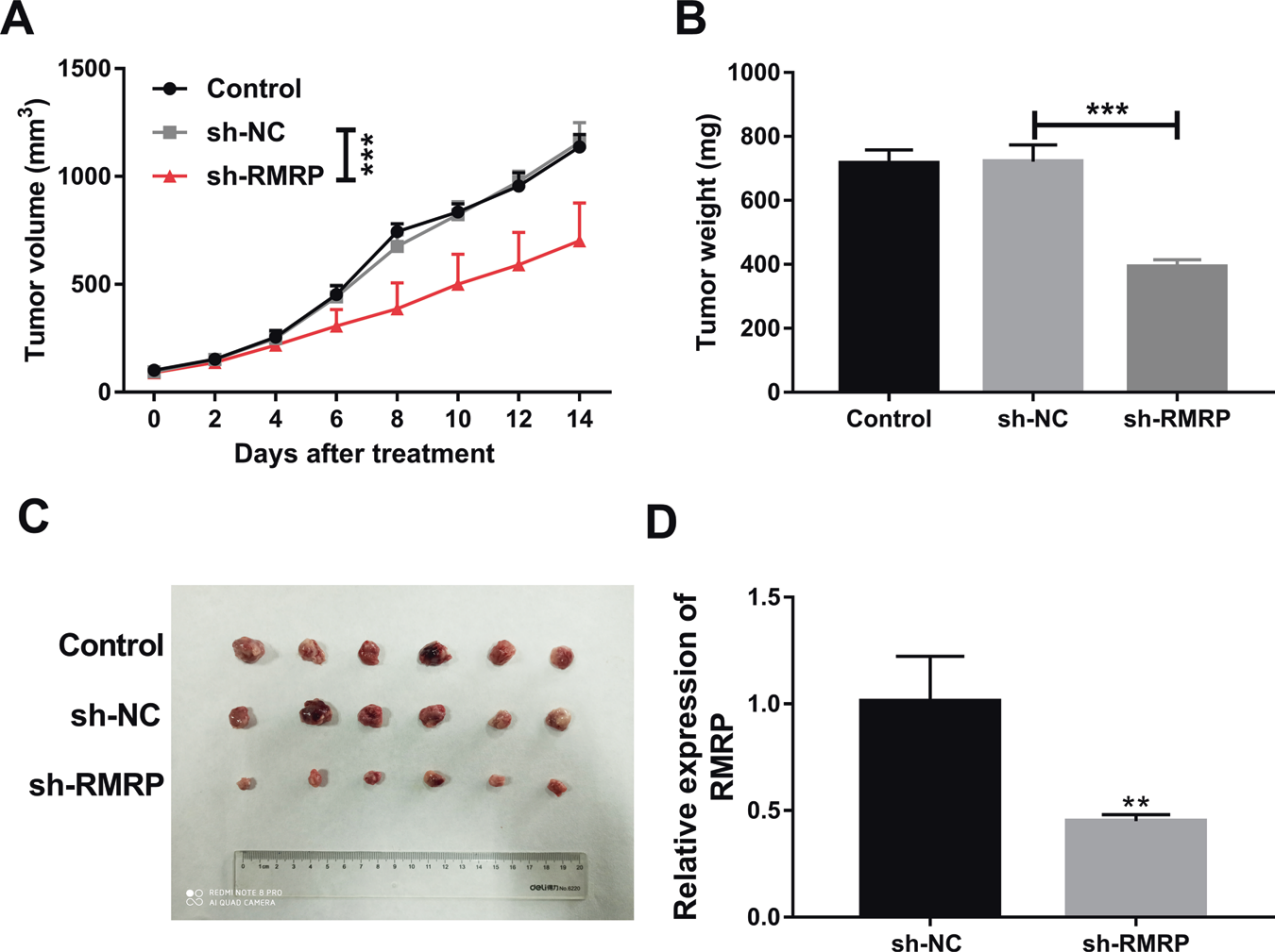
Effects of RMRP knockdown on the growth of TMZ-treated glioma xenograft tumors. **A** The volumes of xenograft tumors were measured every 2 days after TMZ treatment. **B** Tumor weight was determined on day 14 after TMZ treatment. **C** Representative photographs of xenograft tumors in control, sh-NC, and sh-RMRP groups. **D** RMRP level was measured by RT-qPCR assay in xenograft tumors of sh-NC and sh-RMRP groups. Each group contains 6 mice. **P < 0.01. ***P < 0.001.

As shown in Fig. 2D, the IC_50_ values of TMZ were 350.9 and 623.8 µM in U251/TMZ and LN229/TMZ cells, respectively. To reduce TMZ dosage, U251/TMZ cells were used in our animal experiments. In vivo experiments demonstrated that there was a notable reduction of tumor volume and weight in TMZ-treated glioma xenograft tumors derived from U251/TMZ cells infected with sh-RMRP lentiviruses compared to the uninfected group or sh-NC group (Fig. 3A–C). RT-qPCR assay further validated that RMRP level was markedly reduced in glioma xenograft tumors derived from U251/TMZ cells infected with sh-RMRP lentiviruses relative to the sh-NC group (Fig. 3D). That was to say, RMRP knockdown hindered the growth of TMZ-treated glioma xenograft tumors in vivo.

### ZNRF3 was a target of RMRP3

**Fig. 4:**
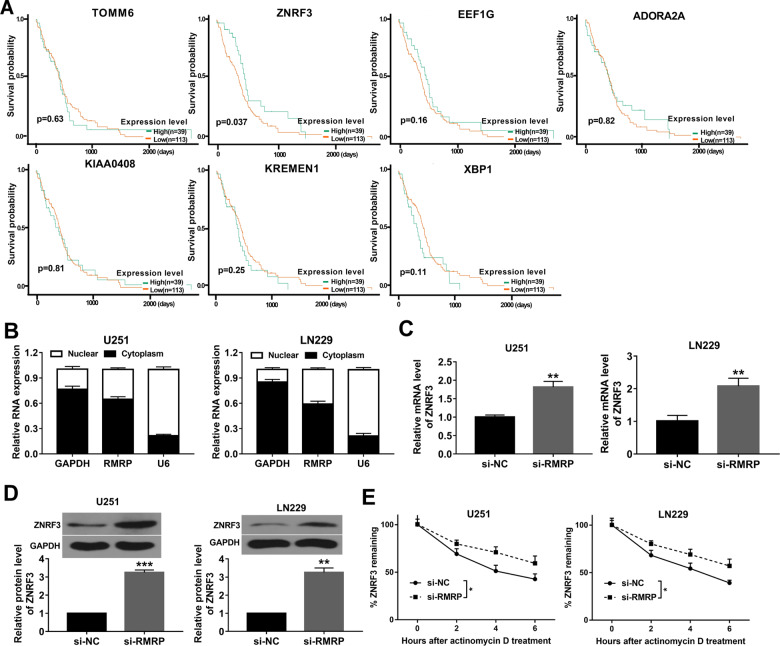
ZNRF3 was a target of RMRP3. **A** Survival analysis of glioma patients with high or low expression of TOMM6, ZNRF3, EEF1G, ADORA2A, KIAA0408, KREMEN1, and XBP1. **B** RMRP level in the nucleus and cytoplasm of U251 and LN229 cells was measured by RT-qPCR assay after the isolation of nuclear and cytoplasmic RNA (*n* = 3). **C**, **D** The effects of RMRP knockdown on ZNRF3 mRNA and protein expression were measured by RT-qPCR and western blot assays at 48 h after transfection in U251 and LN229 cells (*n* = 3). **E** The effect of RMRP knockdown on ZNRF3 mRNA stability was examined through ActD and RT-qPCR assays in U251 and LN229 cells (*n* = 3). **P < 0.01.

Recently, emerging evidence shows that lncRNAs can exert their biological activities through regulating gene expression [24]. To identify genes related to RMRP, gene differential expression analysis was performed for glioma data (*n* = 168) downloaded from the TCGA database and normal brain tissue data (*n* = 150) downloaded from the GTEx database. Moreover, co-expression relationships of RMRP and the top 50 down-regulated genes (Supplementary Table 3) in glioma were predicted through the GEPIA database. Results showed that expression levels of 13 genes were negatively associated with RMRP level (*P* < 0.05) in glioma (Supplementary Table 4). Based on *P-*value (*P* < 0.01), 7 genes, whose expression was highly associated with RMRP level (TOMM6, ZNRF3, EEF1G, ADORA2A, KIAA0408, KREMEN1, XBP1), were pricked out for further explorations. The Ualcan database analysis further revealed that glioma patients with low ZNRF3 expression had a poor prognosis (*P* = 0.037), while the expression of the other 6 genes was not associated with the survival of glioma patients (Fig. 4A). These data suggested the potential regulatory relationships between RMRP and ZNRF3 in glioma. Subcellular localization analyses revealed that RMRP was located in the nuclear and cytoplasm of U251 and LN229 cells (Fig. 4B). In addition, RT-qPCR and western blot assays demonstrated that RMRP depletion led to the remarkable elevation of ZNRF3 mRNA and protein levels in U251 and LN229 cells (Fig. 4C and D). Furthermore, ActD assay showed that RMRP loss enhanced the stability of ZNRF3 mRNA in U251 and LN229 cells (Fig. 4E). In a word, these outcomes showed that RMRP knockdown promoted ZNRF3 expression and improved ZNRF3 mRNA stability in glioma cells.

### ZNRF3 down-regulation weakened the effects of RMRP knockdown on glioma cell proliferation, apoptosis, and TMZ resistance

**Fig. 5:**
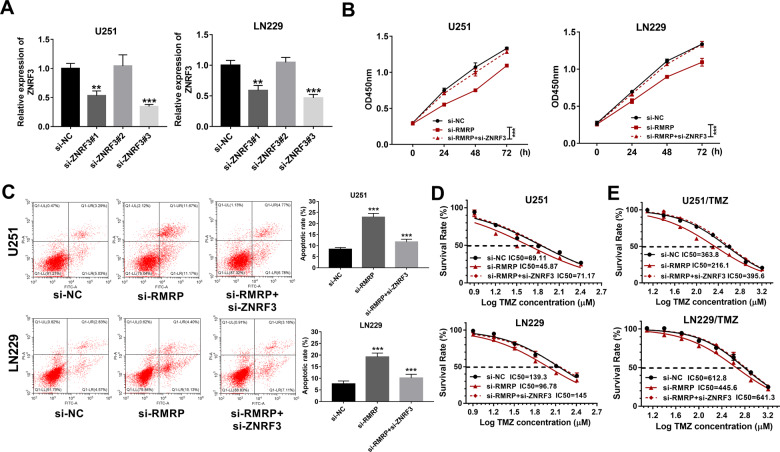
ZNRF3 down-regulation weakened the effects of RMRP knockdown on glioma cell proliferation, apoptosis, and TMZ resistance. **A** Knockdown efficiency analysis of si-ZNRF3#1, si-ZNRF3#2, or si-ZNRF3#3 in U251 and LN229 cells by RT-qPCR assay at 48 h post-transfection (*n* = 3). **B**–**D** U251 and LN229 cells were transfected with si-NC, si-RMRP, or si-RMRP + si-ZNRF3, followed by the measurement of cell proliferative ability **B** (*n* = 3), cell apoptotic rate **C** (*n* = 3), and TMZ IC_50_ values **D** (*n* = 3). **E** TMZ IC_50_ values were determined by CCK-8 assay in U251/TMZ and LN229/TMZ cells treated with different doses of TMZ (*n* = 3). **P < 0.01. ***P < 0.001.

Knockdown efficiency analyses revealed that the transfection of si-ZNRF3#1 or si-ZNRF3#3 led to the notable down-regulation of ZNRF3 mRNA expression level in U251 and LN229 cells (Fig. 5A). Next, si-ZNRF3#3 was used in the following experiments because of its stronger knockdown effect. CCK-8 assay showed that ZNRF3 knockdown markedly abrogated the detrimental effect of RMRP depletion on cell proliferation in U251 and LN229 cells (Fig. 5B). Moreover, ZNRF3 loss triggered a notable reduction in cell apoptotic percentage in U251 cells transfected with si-RMRP (Fig. 5C). Also, cell apoptotic percentage was markedly reduced in si-RMRP-transfected LN229 cells following the introduction of si-ZNRF3 (Fig. 5C). Moreover, ZNRF3 depletion abolished the inhibitory effect of RMRP knockdown on cell survival in TMZ-treated parental glioma cells (U251 and LN229) and TMZ-resistant glioma cells (U251/TMZ and LN229/TMZ) (Figs. 5D and E). Additionally, there was an obvious increase of TMZ IC_50_ value in cells co-transfected with si-RMRP and si-ZNRF3 compared to cells transfected with si-RMRP alone (Figs. 5D and E). In summary, these data revealed that ZNRF3 knockdown weakened the effects of RMRP depletion on glioma cell proliferation, apoptosis, and TMZ resistance. In other words, RMRP knockdown inhibited glioma cell proliferation, facilitated glioma cell apoptosis, and weakened the resistance of glioma cells to TMZ through up-regulating ZNRF3.

### RMRP regulated ZNRF3 expression and mRNA stability through IGF2BP3

**Fig. 6:**
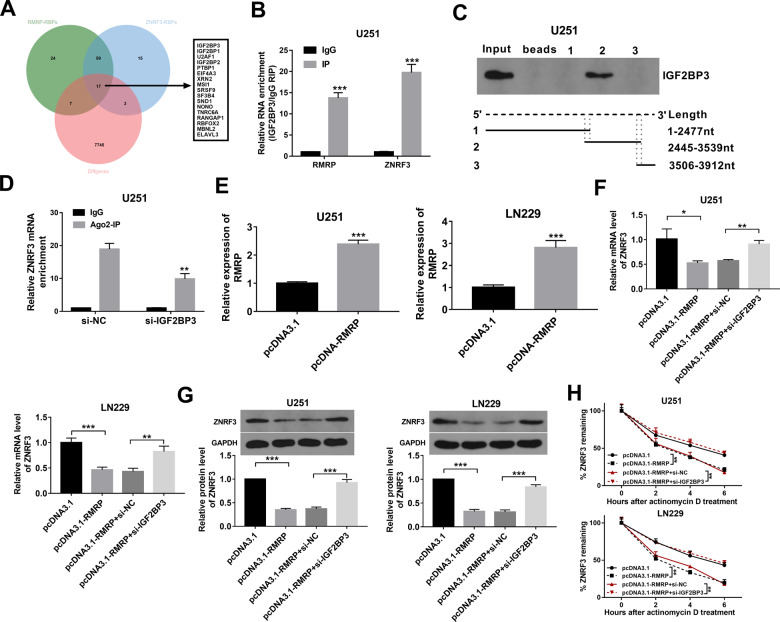
RMRP regulated ZNRF3 expression and ZNRF3 mRNA stability through RBP IGF2BP3. **A** Venn analysis for RMRP-RBPs, ZNRF3-RBPs, and differentially expressed genes in glioma versus normal tissues. RMRP-RBPs (green): RBPs with the possibility to bind with RMRP (predicted by the POSTAR database). ZNRF3-RBPs (blue): RBPs with the possibility to bind with ZNRF3 mRNA (predicted by the POSTAR database). Diffgenes (red): Differentially expressed genes in glioma versus normal brain tissues (data were downloaded from the TCGA/GTEx databases). **B** RIP assay was performed using IgG or IGF2BP3 antibody in U251 cells. Next, RMRP and ZNRF3 mRNA levels enriched by IgG or IGF2BP3 antibodies were measured by RT-qPCR assay (*n* = 3). **C** RNA pull-down assay was performed using biotin-labeled ZNRF3 mRNA 3’UTR fragment 1, fragment 2, and fragment 3 in U251 cells, followed by the detection of IGF2BP3 protein level via western blot assay. **D** U251 cells were transfected with si-NC or si-IGF2BP3. At 48 h after transfection, a RIP assay was performed using IgG or Ago2 antibody. **E** U251 and LN229 cells were transfected with pcDNA3.1 or pcDNA3.1-RMRP. Forty-eight hours later, the RMRP level was measured by RT-qPCR assay (*n* = 3). **F**, **G** U251 and LN229 cells were transfected with pcDNA3.1, pcDNA3.1-RMRP, pcDNA3.1-RMRP + si-NC, or pcDNA3.1-RMRP + si-IGF2BP3. **F**, **G** ZNRF3 mRNA and protein levels were determined by RT-qPCR and western blot assays (*n* = 3). **H** ZNRF3 mRNA stability was tested by Actinomycin D and RT-qPCR assays (*n* = 3). **P < 0.01. ***P < 0.001.

Recently, emerging studies show that RNA binding proteins (RBPs) with the binding activities with multiple RNA molecules including mRNAs and non-coding RNAs play vital roles in regulating RNA metabolism and tumor development [25–27]. Thus, RBPs that could bind with RMRP and ZNRF3 mRNA were screened out through the POSTAR database. Combined with the aforementioned TCGA/GTEx differential gene expression data, 17 RBPs with the possibility to interact with both RMRP and ZNRF3 mRNA were found to be differentially expressed in glioma (Fig. 6A, Supplementary Table 5). Among these RBPs, IGF2BP3 was picked out for further examinations given its noticeably high expression in glioma tissues (Supplementary Table 5). RIP assay coupled with RT-qPCR assay demonstrated that RMRP and ZNRF3 mRNA could be substantially enriched by the IGF2BP3 antibody in U251 cells (Fig. 6B). Considering the central role of the 3’ non-coding region (3’UTR) region in determining mRNA stability, the essential sites in controlling the binding activity of IGF2BP3 and ZNRF3 mRNA in the 3’UTR were further examined. POSTAR2 database revealed that there were 5 potential IGF2BP3 binding sites in the 3’UTR of ZNRF3 mRNA. These binding sites were presented in Supplementary Table 6. RNA pull-down assay showed that IGF2BP3 protein could be largely pulled down by biotin-labeled ZNRF3 mRNA 3’UTR fragment 2 (2445–3539 nt), but could not be pulled down by biotin-labeled ZNRF3 mRNA 3’UTR fragment 1 (1-2477 nt) and fragment 3 (3506-3912 nt) (Fig. 6C). Because ZNRF3 mRNA 3’UTR fragment 1 contains IGF2BP3 binding site 1 and 2, fragment 2 contains binding site 2-4, fragment 3 covers the binding site 4 and 5, we believed that IGF2BP3 binding site 3 in ZNRF3 mRNA 3’UTR (chr22: 29056929-29056950, GCTTTTAAAATGAGGTCTAAAA) might mainly govern the binding activity of ZNRF3 mRNA 3’UTR and IGF2BP3 protein. The m6A2Target prediction analysis suggested that IGF2BP3 protein could bind with ZNRF3 mRNA in an m6A-independent manner (http://m6a2target.canceromics.org/#/binddetail/Human/IGF2BP3/ZNRF3/HEK293T). Recently, some studies showed that IGF2BP3 could regulate gene expression by altering the interaction of mRNAs and RNA-induced silencing complex (RISC) [28, 29]. Thus, we supposed that IGF2BP2 might influence ZNRF3 expression through a RISC-dependent mechanism. Given the vital role of Argonaute 2 (Ago2) in RISC, the effect of IGF2BP3 knockdown on the interaction of Ago2 (a core component of RISC) and ZNRF3 was examined in our project. Results showed that IGF2BP3 loss led to the notable reduction of ZNRF3 level enriched by Ago2 antibody in U251 cells (Fig. 6D), suggesting that IGF2BP3 knockdown weakened the interaction of ZNRF3 and RISC in glioma cells. Next, overexpression efficiency analyses showed that the transfection of pcDNA3.1-RMRP overexpression plasmid markedly improved RMRP expression level in U251 and LN229 cells compared to the pcDNA3.1 control group (Fig. 6E). Moreover, RT-qPCR and western blot assays demonstrated that RMRP overexpression led to the notable reduction in ZNRF3 mRNA and protein levels in U251 and LN229 cells (Fig. 6F and G). Additionally, ectopic expression of RMRP reduced ZNRF3 mRNA stability in U251 and LN229 cells (Fig. 6H). Furthermore, IGF2BP3 knockdown markedly alleviated the detrimental effects of RMRP on ZNRF3 expression and mRNA stability in U251 and LN229 cells (Fig. 6F–H).

### RMRP knockdown reduced β-catenin expression by up-regulating ZNRF3 in glioma cells

**Fig. 7:**
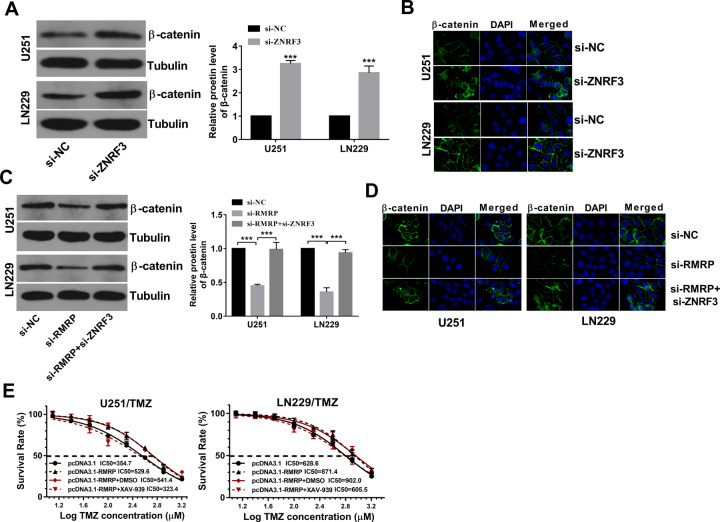
RMRP knockdown inhibited β-catenin expression by up-regulating ZNRF3 in glioma cells. **A**, **B** Cells were transfected with si-NC or si-ZNRF3 for 48 h, followed by the measurement of β-catenin protein level via western blot assay **A** (*n* = 3) and IF assay (*n* = 1) **B**. **C**, **D** Cells were transfected with si-NC, si-RMRP, or si-RMRP + si-ZNRF3. At 48 h post-transfection, western blot assay **C** (*n* = 3) and IF assay **D** (*n* = 1) were performed to examine the protein expression pattern of β-catenin. **E** U251/TMZ and LN229/TMZ cells were transfected with pcDNA3.1 or pcDNA3.1-RMRP for 24 h and then stimulated with or without DMSO/XAV-939 (10 µM) and different doses of TMZ for 24 h, followed by the measurement of TMZ IC_50_ values via CCK-8 assay (*n* = 3).

Next, the western blot assay showed that ZNRF3 loss led to the notable increase of β-catenin protein level in U251 and LN229 cells (Fig. 7A). Also, enhanced β-catenin protein signals were observed in U251 and LN229 cells transfected with si-ZNRF3 compared with si-NC-transfected cells (Fig. 7B). In addition, western blot and IF assays showed that RMRP silence reduced β-catenin protein expression and attenuated β-catenin signal intensity, while ZNRF3 down-regulation markedly alleviated the inhibitory effect of RMRP loss on β-catenin expression in U251 and LN229 cells (Fig. 7C and D). Moreover, the introduction of Wnt/β-catenin signaling pathway inhibitor XAV-939 abrogated RMRP-mediated TMZ resistance and inhibited the increase of cell survival rate induced by RMRP in U251/TMZ and LZ229/TMZ cells (Fig. 7E).

### β-catenin facilitated RMRP transcription by transcription factor 4 (TCF4) in glioma cells

Additionally, a previous study suggested that TCF4 might participate in mediating the regulatory effect of β-catenin on RMRP in HEK293 cells [30]. TCF4 is a member of the T-cell factor/lymphoid enhancer-binding factor (TCF/LEF) transcription factor family, which has been reported to be the major downstream effectors of the Wnt/β-catenin signaling pathway [31–33]. Thus, we further investigated whether β-catenin could regulate lncRNA RMRP expression by TCF4 in glioma cells.

**Fig. 8:**
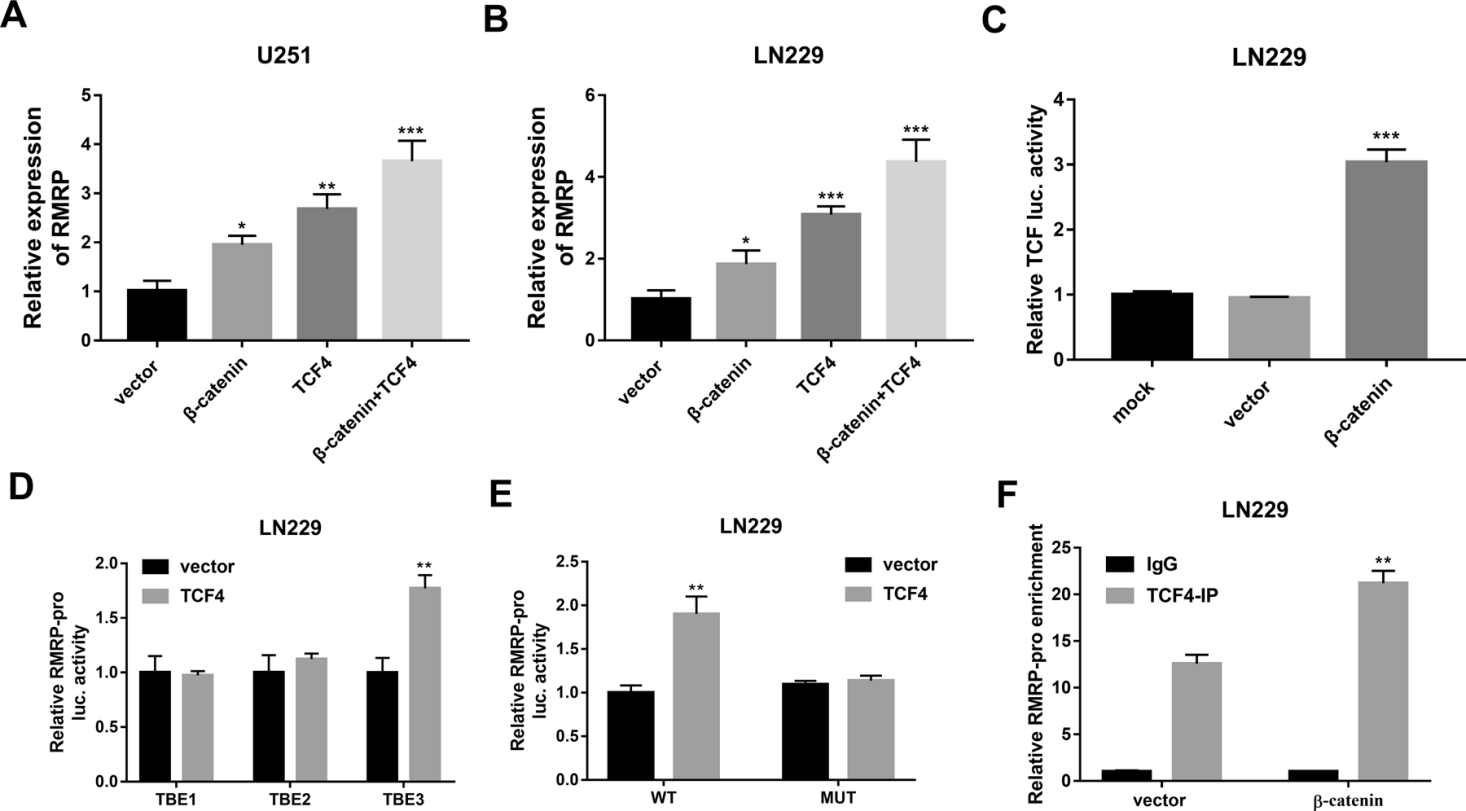
β-catenin facilitated RMRP transcription by TCF4 in glioma cells. **A**, **B** U251 and LN229 cells were transfected with pcDNA3.1 empty vector, pcDNA-β-catenin, pcDNA-TCF4, or pcDNA-β-catenin+pcDNA-TCF4. Forty-eight hours later, the RMRP level was examined by RT-qPCR assay (*n* = 3). **C** LN229 cells were transfected with TCF/LEF1-Luc reporter, TCF/LEF1-Luc reporter+pcDNA3.1 empty vector, or TCF/LEF1-Luc reporter+ pcDNA-β-catenin, followed by the detection of luciferase activity at 48 h after transfection. **D** LN229 cells were co-transfected with pRL-TK Renilla luciferase plasmid, pGL3-luc-TBE1/pGL3-luc-TBE2/pGL3-luc-TBE3 and pcDNA3.1-TCF4 plasmid/pcDNA3.1 empty vector. At 48 h post-transfection, luciferase activities were measured. **E** LN229 cells were co-transfected with pRL-TK Renilla luciferase plasmid, pcDNA3.1-TCF4 plasmid/pcDNA3.1 empty vector, and pGL3-luc-TBE3(WT)/pGL3-luc-TBE(MUT) reporter. Forty-eight hours later, luciferase activities were measured. **F** LN229 cells were transfected with pcDNA3.1 empty vector or pcDNA-β-catenin. Forty-eight hours later, a CHIP assay was carried out using TCF4 or IgG antibody. The level of RMRP gene fragment enriched by IgG or TCF4 antibody was measured by PCR. **P < 0.01. ***P < 0.001.

Our data disclosed that β-catenin and TCF4 overexpression could lead to a notable increase of RMRP expression in U251 and LN229 cells (Fig. 8A and B). Moreover, there was a higher RMRP expression level in U251 and LN229 cells co-transfected with β-catenin and TCF4 than in cells transfected with β-catenin or TCF4 alone (Fig. 8A and B). These data suggested that β-catenin could function as a coactivator of TCF4 to induce RMRP expression. Additionally, β-catenin overexpression notably increased the luciferase activities of TCF/LEF1-Luc reporter containing TCF/LEF1 response elements in U251 cells (Fig. 8C), suggesting that β-catenin could induce gene transcription activation by binding with TCF/LEF1 binding elements. Hence, TCF4 binding elements in the 2000-bp promoter sequences upstream of the RMRP transcription start site were predicted by the JASPAR website (http://jaspar.genereg.net/). Results showed that there were 3 potential TCF4 binding elements in the promoter sequences of RMRP (Relative profile score threshold: 90%). The promoter sequences of RMRP and the predicted TCF4 binding sites in the sequences (marked in bold) were shown in supplementary file 1. Next, RMRP gene promoter fragment containing putative TCF4 binding site 1, 2, or 3 was constructed into the pGL3-Luc reporter, and generated recombinant plasmid was named pGL3-luc-TBE1, pGL3-luc-TBE2, or pGL3-luc-TBE3, respectively. Subsequent luciferase assay further demonstrated that TCF4 overexpression markedly enhanced the luciferase activities of the pGL3-luc-TBE3 reporter containing TBE3 (TCF4 binding site 3), but did not influence the luciferase activities of a pGL3-luc-TBE1 reporter containing TBE1 (TCF4 binding site 1) or pGL3-luc-TBE2 reporter containing TBE2 (TCF4 binding site 2) in U251 cells (Fig. 8D). These data suggested that TCF4 could activate the transcription of RMRP by interacting with the binding site 3. To further validate the vital role of TBE3 in TCF4-mediated transcription activation of RMRP promoter, pGL3-luc-TBE3 (MUT) reporter with mutant TCF4 binding site 3 was constructed. Luciferase reporter assay showed that the mutation of TCF4 binding site 3 abrogated TCF4-mediated transcription activation at the RMRP promoter in U251 cells (Fig. 8E). Additionally, the CHIP assay also demonstrated that TCF4 could bind to the promoter region of RMRP, and β-catenin overexpression further potentiated the binding activity of TCF4 and the RMRP promoter region (Fig. 8F). Taken together, these data suggested that β-catenin could activate RMRP transcription by TCF4.

## Discussion

In this project, our data revealed that RMRP was highly expressed in glioma tissues and RMRP knockdown inhibited glioma cell proliferation and promoted glioma cell apoptosis, which was in accordance with a previous study [21]. Moreover, we demonstrated that RMRP depletion reduced the resistance of glioma cells to TMZ in vitro and inhibited the growth of TMZ-treated glioma xenograft tumors in vivo. This is the first study to demonstrate that RMRP knockdown could weaken TMZ resistance in glioma.

Next, ZNRF3 was screened out based on differential expression and co-expression analyses for glioma and normal brain tissue data in the TCGA and GTEx databases and bioinformatics analysis. Differential expression analysis revealed that ZNRF3 expression was notably down-regulated in glioma tissues versus normal tissues. Co-expression analysis showed that ZNRF3 expression level was negatively associated with RMRP expression in glioma tissues. Moreover, bioinformatics analysis by the Ualcan database disclosed that glioma patients with low ZNRF3 expression had a poor prognosis. These data suggested that there was a potential regulatory relationship between RMRP and ZNRF3. Moreover, ZNRF3 has been identified to be a potential tumor suppressor in several cancers such as gastric cancer [34], nasopharyngeal cancer [35], and esophageal squamous cell cancer [36]. For instance, enforced expression of ZNRF3 inhibited cell proliferation and facilitated cell apoptosis in colorectal cancer [37]. To our knowledge, the roles of ZNRF3 in glioma progression and TMZ resistance along with related molecular mechanisms are not or poorly characterized. Thus, we further investigated whether RMRP could regulate ZNRF3 expression in glioma. Our outcomes disclosed that RMRP knockdown led to the remarkable increase of ZNRF3 mRNA and protein expression and ZNRF3 mRNA stability in glioma cells. Additionally, ZNRF3 downregulation markedly weakened the effects of RMRP knockdown on glioma cell proliferation, apoptosis, and TMZ resistance.

Considering the potential binding activity of RBPs and mRNAs/non-coding RNAs and the vital roles of RBPs in regulating RNA fate and tumor progression, RBP IGF2BP3 that could bind with both RMRP and ZNRF3 mRNA was screened out for further investigations because of the notably high expression of IGF2BP3 in glioma. IGF2BP3, also named IMP3, is a member of the insulin-like growth factor-2 mRNA-binding protein family [38]. IGF2BP3 has been reported to be involved in the regulation of tumor cell fate (*e.g*. growth, survival, invasion, drug resistance) in multiple cancers [38, 39]. For instance, IGF2BP3 knockdown hindered glioma cell proliferation and cell cycle progression and inhibited glioma stem-like cell migration [40, 41]. Moreover, glioma patients with a higher IGF2BP3 expression level had a shorter overall and disease-free survival, and IGF2BP3 expression was positively associated with glioma grades [42, 43]. It has been reported that IGF2BP3 can regulate gene expression and tumor progression by multiple mechanisms [44]. One common mechanism is that IGF2BP3 can function as an N6-methyladenosine (m6A) reader to regulate gene expression in an m6A-dependent manner [45, 46]. For instance, IGF2BP3 facilitated TMBIM6 expression and increased TMBIM6 mRNA stability by reading the m6A modification of TMBIM6 mRNA in laryngeal squamous cell cancer [47]. IGF2BP3 could directly interact with the m6A-modified region on ATP-binding cassette transporters subfamily B member 1 (ABCB1) mRNA, resulting in the high expression of ABCB1 and the elevation of ABCB1 mRNA stability in colorectal cancer cells [48]. Recently, some studies suggested that IGF2BP3 could inhibit or promote target gene expression through a microRNA- or RNA-induced silencing complex (RISC)-dependent mechanism [29, 44, 49]. For instance, IGF2BP3 knockdown triggered the notable increase of DCBLD2, ZFP36L1, and CLDN1 mRNA levels, and reduced the interactions of these mRNAs and RISC complex [49]. IGF2BP3 reduced zonula occludens-1 (ZO-1) expression by facilitating the formation of a miR-191-5p-induced RISC in hepatocellular carcinoma cells [50]. The prediction analysis by the m6A2Target database suggested that IGF2BP3 protein could bind with ZNRF3 mRNA through an m6A-independent mechanism. Moreover, our data showed that IGF2BP3 could bind with ZNRF3 3’non-coding region (3’UTR) and IGF2BP3 knockdown weakened the interaction of ZNRF3 and RISC. These data suggested that IGF2BP3 might regulate ZNRF3 expression in a RISC-dependent mechanism. Moreover, we demonstrated that RMRP inhibited ZNRF3 expression and reduced ZNRF3 mRNA stability by IGF2BP3.

ZNRF3, a membrane E3 ligase, also has been found to be a negative regulator of the Wnt signaling pathway [51]. The Wnt/β-catenin pathway has been found to be aberrantly activated in plenty of cancers and to be involved in the regulation of cancer progression [52, 53]. Also, previous studies showed that the activation of the Wnt signaling pathway could enhance the resistance of cancer cells to chemotherapeutic drugs [54]. Moreover, it has been reported that the Wnt/β‐catenin signaling pathway is closely linked with the tumorigenesis and progression of glioma and participates in the regulation of TMZ resistance in glioma [55]. Hence, we further investigated whether the RMRP/ZNRF3 axis could regulate the Wnt/β‐catenin signaling pathway in glioma cells. Our results showed that ZNRF3 knockdown promoted β‐catenin expression in glioma cells. Moreover, RMRP loss inhibited β-catenin expression by up-regulating ZNRF3 in glioma cells. Additionally, the inhibition of the Wnt/β‐catenin signaling pathway by XAV-939 weakened RMRP-induced TMZ resistance in glioma cells. Furthermore, β-catenin increase markedly facilitated RMRP expression by TCF4 and TCF4 promoted RMRP expression by directly binding with the RMRP promoter region in glioma cells. Recently, Battistelli et al. designed a HOTAIR deletion mutant form that could effectively counteract lncRNA HOTAIR activity and oncogenic roles [56]. Thus, we supposed that an RMRP mutant with the depletion of the IGF2BP3-binding domain might be a potential RNA-based strategy to counteract glioma progression and drug resistance in glioma.

## Conclusions

Taken together, our data demonstrated that RMRP was highly expressed in glioma tissues versus normal brain tissues. RMRP depletion hindered cell proliferation, induced cell apoptosis, and reduced TMZ resistance in glioma cells, and curbed the growth of TMZ-treated glioma xenograft tumors in vivo, hinting that RMRP loss or inhibition could be used to mitigate TMZ resistance. Molecular mechanism investigations revealed that RMRP exerted its functions by reducing ZNRF3 expression and ZNRF3 mRNA stability via IGF2BP3 in glioma. Moreover, we demonstrated that RMRP/ZNRF3 axis and Wnt/β‐catenin signaling pathway could form a positive feedback loop to regulate TMZ resistance in glioma. To our knowledge, this is the first study to test the effect of RMRP on TMZ resistance in glioma and elucidate the molecular mechanisms of RMRP underlying glioma development and TMZ resistance. Given the vital roles of ZNRF3 in Wnt/β‐catenin signaling and cancer progression, further investigations on ZNRF3 regulatory mechanisms can deepen our understanding of the pathogenesis of glioma. The high expression of RMRP in glioma tumor tissues and close association of RMRP and glioma development suggests the potential values of RMRP in the diagnosis and treatment of glioma. Moreover, our data showed that RMRP overexpression could induce the sustained activation of the Wnt/β-catenin signaling pathway, while the sustained activation of the Wnt/β-catenin pathway has been found to be closely linked with tumor initiation, progression, recurrence, immune escape, and therapeutic resistance [57]. The information suggests the clinical significance of RMRP in tumor initiation, progression, recurrence, immune escape, and therapeutic resistance.

## Supplementary information

Supplementary meterials

WB data

## Data Availability

The data and material presented in this manuscript is available from the corresponding author on reasonable request.
