# Supplementary material for: A positive feedback loop of lncRNA-RMRP/ZNRF3 axis and Wnt/β-catenin signaling regulates the progression and temozolomide resistance in glioma
Source: Cell Death Dis. 2021 Oct 16;12(11):952. doi: 10.1038/s41419-021-04245-y (PMC8520527; doi:10.1038/s41419-021-04245-y)
Supplement: Supplementary file 1 — Supplementary meterials [file 41419_2021_4245_MOESM1_ESM.pdf]

Supplementary Table 1 Sense siRNA sequences of si-NC, si-RMRP#1, si-RMRP#2, si-RMRP#3, si-ZNRF3#1, si-ZNRF3#2, si-ZNRF3#3, si-IGF2BP3#1, si-IGF2BP3#2 and si-IGF2BP3#3

si-NC	sense, 5’- UUCUCCGAACGUGUCACGUTT -3′
si-RMRP#1	sense, 5′- CCUAGGCUACACACUGAGGACUTT -3′
si-RMRP#2	sense, 5′- CGUCAGCUCCCUCUAGUUACGTT -3′
si-RMRP#3	sense, 5′- GCCUGUAUCCUAGGCUACAUTT -3′
si-ZNRF3#1	sense, 5′-GAAUAUUUAUUUACUAAUAUA -3′
si-ZNRF3#2	sense, 5′- GAUAUUGUAUGUAAAUGUAAA -3′
si-ZNRF3#3	sense, 5′- GCUGCUACACUGAGGACUATT -3′
si-IGF2BP3#1	sense, 5′- AUAAAGUAUACAUUCUCACAG -3′
si-IGF2BP3#2	sense, 5′- GAUGAUCACAGUCAUGUUACA -3′
si-IGF2BP3#3	sense, 5′- GCUAAGAAAUAAUUCUAUAAU -3′

Supplementary Table 2 Quantitative PCR primer sequences for GAPDH, RMRP, HOXA-AS3, CASC9, and ZNRF3.

GAPDH	F: 5'- GAAGGTGAAGGTCGGAGTC -3'
	R: 5'- GAAGATGGTGATGGGATTTC -3'
lncRNA RMRP	F: 5'- ACTCCAAAGTCCGCCAAGA -3'
	R:5'- TGCGTAACTAGAGGGAGCTGAC -3′
lncRNA HOXA-AS3	F: 5′- ACAAGTAGACAGGGCACTCG -3′
	R:5′- GGCTGGAAAGGTCGGTTGTA -3′
lncRNA CASC9	F: 5′- TTGGTCAGCCACATTCATGGT -3′
	R:5′- AGTGCCAATGACTCTCCAGC -3′
ZNRF3	F: 5′- GCGGGTCATCCCCTGTAC -3′
	R:5′- GCTTGGGTTTCCCTTTTGTT -3′

Supplementary Table 3 Top 50 down-regulated genes in glioma tissues (n=168) versus normal brain tissues (n=150).

gene_id	gene_name	gene_type	baseMean	log2FoldChange
ENSG00000214736.7	TOMM6	protein_coding	1374.204	-13.9316
ENSG00000183762.12	KREMEN1	protein_coding	1168.519	-13.6978
ENSG00000213578.6	CPLX3	protein_coding	881.104	-13.1532
ENSG00000100219.16	XBP1	protein_coding	650.0047	-12.8518
ENSG00000243509.6	TNFRSF6B	protein_coding	563.3238	-12.6451
ENSG00000213689.14	TREX1	protein_coding	394.5534	-12.1309
ENSG00000145863.11	GABRA6	protein_coding	1263.159	-11.0768
ENSG00000103310.10	ZP2	protein_coding	259.5472	-10.6913
ENSG00000254772.10	EEF1G	protein_coding	10691.19	-10.0734
ENSG00000139574.8	NPFF	protein_coding	70.47618	-9.64633
ENSG00000187664.9	HAPLN4	protein_coding	788.5805	-9.46491
ENSG00000204983.14	PRSS1	protein_coding	66.5347	-9.14992
ENSG00000259803.7	SLC22A31	protein_coding	1122.359	-9.14244
ENSG00000161992.6	PRR35	protein_coding	905.7517	-9.08997
ENSG00000140488.16	CELF6	protein_coding	842.5066	-8.94603
ENSG00000254852.8	NPIPA2	protein_coding	41.43677	-8.7939

ENSG00000275896.5	PRSS2	protein_coding	59.96191	-8.51508
ENSG00000169347.16	GP2	protein_coding	44.80562	-8.47527
ENSG00000140506.17	LMAN1L	protein_coding	222.1373	-8.46198
ENSG00000183336.8	BOLA2	protein_coding	30.36661	-8.29844
ENSG00000124493.13	GRM4	protein_coding	5095.998	-8.26487
ENSG00000175535.6	PNLIP	protein_coding	41.79157	-8.25373
ENSG00000086570.12	FAT2	protein_coding	4351.549	-8.17583
ENSG00000183379.9	SYNDIG1L	protein_coding	5193.95	-8.1221
ENSG00000115665.9	SLC5A7	protein_coding	70.7648	-8.01853
ENSG00000128271.22	ADORA2A	protein_coding	1143.007	-7.97506
ENSG00000189367.15	KIAA0408	protein_coding	2062.133	-7.9244
ENSG00000243789.11	JMJD7	protein_coding	563.5629	-7.89467
ENSG00000272573.6	MUSTN1	protein_coding	302	-7.82681
ENSG00000213927.4	CCL27	protein_coding	22.78999	-7.82074
ENSG00000101200.5	AVP	protein_coding	92.97653	-7.80529
**ENSG00000183579.16**	**ZNRF3**	**protein_coding**	**973.3927**	**-7.79792**
ENSG00000188038.8	NRN1L	protein_coding	69.08776	-7.77971
ENSG00000142789.20	CELA3A	protein_coding	24.23641	-7.7586
ENSG00000100249.4	C22orf31	protein_coding	15.80291	-7.48736
ENSG00000170477.13	KRT4	protein_coding	43.84427	-7.34327
ENSG00000111245.15	MYL2	protein_coding	59.22261	-7.33423
ENSG00000137843.12	PAK6	protein_coding	1470.04	-7.32924
ENSG00000171195.11	MUC7	protein_coding	21.18084	-7.28611
ENSG00000094796.5	KRT31	protein_coding	182.3399	-7.22796
ENSG00000171772.16	SYCE1	protein_coding	372.6	-7.20241
ENSG00000243708.10	PLA2G4B	protein_coding	1990.801	-7.19196
ENSG00000204001.9	LCN8	protein_coding	206.1484	-7.14751
ENSG00000118271.10	TTR	protein_coding	653.9319	-7.08388
ENSG00000182450.13	KCNK4	protein_coding	294.6109	-7.00745
ENSG00000143032.8	BARHL2	protein_coding	258.2312	-6.97137
ENSG00000102924.12	CBLN1	protein_coding	3189.46	-6.83188
ENSG00000196689.12	TRPV1	protein_coding	569.4355	-6.81289
ENSG00000214338.10	SOGA3	protein_coding	353.8444	-6.79365

Supplementary Table 4 Correlation analyses of RMRP level and the top 50 down-regulated genes via GEPIA database.

gene_id	gene_name	gene_type	R value	P-value
ENSG00000214736.7	TOMM6	protein_coding	-0.29	0.00015
ENSG00000183579.16	ZNRF3	protein_coding	-0.29	0.00019
ENSG00000254772.10	EEF1G	protein_coding	-0.22	0.0055
ENSG00000128271.22	ADORA2A	protein_coding	-0.21	0.007
ENSG00000189367.15	KIAA0408	protein_coding	-0.21	0.0076
ENSG00000183762.12	KREMEN1	protein_coding	-0.2	0.0098
ENSG00000100219.16	XBP1	protein_coding	-0.2	0.0088
ENSG00000243789.11	JMJD7	protein_coding	-0.2	0.012
ENSG00000213689.14	TREX1	protein_coding	-0.19	0.017
ENSG00000254852.8	NPIPA2	protein_coding	-0.19	0.015
ENSG00000214338.10	SOGA3	protein_coding	-0.17	0.032
ENSG00000188038.8	NRN1L	protein_coding	-0.16	0.048
ENSG00000137843.12	PAK6	protein_coding	-0.16	0.041

ENSG00000183379.9	SYNDIG1L	protein_coding	-0.15	0.054
ENSG00000272573.6	MUSTN1	protein_coding	-0.15	0.055
ENSG00000140488.16	CELF6	protein_coding	-0.14	0.068
ENSG00000187664.9	HAPLN4	protein_coding	-0.12	0.13
ENSG00000243708.10	PLA2G4B	protein_coding	-0.12	0.12
ENSG00000204001.9	LCN8	protein_coding	-0.11	0.16
ENSG00000182450.13	KCNK4	protein_coding	-0.11	0.17
ENSG00000171772.16	SYCE1	protein_coding	-0.1	0.18
ENSG00000196689.12	TRPV1	protein_coding	-0.097	0.22
ENSG00000243509.6	TNFRSF6B	protein_coding	-0.082	0.3
ENSG00000145863.11	GABRA6	protein_coding	-0.07	0.38
ENSG00000175535.6	PNLIP	protein_coding	-0.067	0.39
ENSG00000213927.4	CCL27	protein_coding	-0.065	0.41
ENSG00000213578.6	CPLX3	protein_coding	-0.064	0.42
ENSG00000171195.11	MUC7	protein_coding	-0.057	0.47
ENSG00000118271.10	TTR	protein_coding	-0.056	0.48
ENSG00000183336.8	BOLA2	protein_coding	-0.052	0.51
ENSG00000100249.4	C22orf31	protein_coding	-0.05	0.52
ENSG00000115665.9	SLC5A7	protein_coding	-0.047	0.55
ENSG00000169347.16	GP2	protein_coding	-0.045	0.59
ENSG00000139574.8	NPFF	protein_coding	-0.044	0.58
ENSG00000101200.5	AVP	protein_coding	-0.043	0.58
ENSG00000124493.13	GRM4	protein_coding	-0.041	0.61
ENSG00000204983.14	PRSS1	protein_coding	-0.04	0.64
ENSG00000170477.13	KRT4	protein_coding	-0.04	0.62
ENSG00000111245.15	MYL2	protein_coding	-0.04	0.54
ENSG00000094796.5	KRT31	protein_coding	-0.039	0.62
ENSG00000161992.6	PRR35	protein_coding	-0.038	0.63
ENSG00000259803.7	SLC22A31	protein_coding	-0.036	0.65
ENSG00000140506.17	LMAN1L	protein_coding	-0.031	0.69
ENSG00000143032.8	BARHL2	protein_coding	-0.021	0.79
ENSG00000086570.12	FAT2	protein_coding	-0.01	0.9
ENSG00000103310.10	ZP2	protein_coding	0.0035	0.96
ENSG00000102924.12	CBLN1	protein_coding	0.018	0.82
ENSG00000275896.5	PRSS2	protein_coding	0.025	0.75
ENSG00000142789.20	CELA3A	protein_coding	0.16	0.044

Supplementary Table 5 Expression patterns of 17 differentially expressed RBPs that had the likelihood to bind with both RMRP and ZNRF3 mRNA.

gene_name	gene_type	baseMean	log2FoldChange
IGF2BP3	protein_coding	309.1855	7.126539
IGF2BP1	protein_coding	13.30504	4.005563
U2AF1	protein_coding	39.64123	2.069723
IGF2BP2	protein_coding	572.0013	1.969356
PTBP1	protein_coding	6228.647	1.66138
EIF4A3	protein_coding	2968.521	1.458592
XRN2	protein_coding	3305.495	1.37398
MSI1	protein_coding	1341.736	1.359946
SRSF9	protein_coding	2870.31	1.304158

SF3B4	protein_coding	2578.165	1.203024
SND1	protein_coding	6167.769	1.1116
NONO	protein_coding	13010.02	1.086084
TNRC6A	protein_coding	4124.074	-1.06001
RANGAP1	protein_coding	6934.024	-1.21977
RBFOX2	protein_coding	6642.03	-1.58743
MBNL2	protein_coding	4176.68	-1.6651
ELAVL3	protein_coding	7141.248	-2.37595

Supplementary Table 6 Putative binding sites between IGF2BP3 and ZNRF3 3'UTR by POSTAR2 database.

RBP	RBP info	Tissue type	Position	Genomic context
IGF2BP3	info	HEK293	chr22:29055236-29055286	UTR3
IGF2BP3	info	HEK293	chr22:29056067-29026099	UTR3
IGF2BP3	info	HEK293	chr22:29056929-29056950	UTR3
IGF2BP3	info	HEK293	chr22:29057129-29057161	UTR3
IGF2BP3	info	HEK293	chr22:29057294-29057273	UTR3

Supplementary file 1 The 2000 bp promoter sequence upstream of the RMRP transcription start site and potential JASPAR-predicted TCF4 binding site (marked in bold) in the promoter sequence (Relative profile score threshold: 90%).

CAAAAATTAGCCGGGCGTGGT**GGCAGGTGCC**TGTAATCCCAGTTACTTGAGAGGCTG
AGGCAGGAGAATCGCTTGAACCTGGGAGCCAGAGGTTGCAGTGAGCCGAGATTGCGC
CGCTGCACTCCAGCCTGGGTGACAGAGCGAGACTCCATCTCGAAAAAAACAAAAACT
TTGGTTGTTAGACAGTTCATGCACAAAGCCAAAATTTGTCCTTTATGTATCTTCTTTCCA
TAGTGCTTACTGGAGCCTTCCAAAATAATGTCTCCTCAAGGTGACAGCCCCTCAGGAAT
TTGAAGGCAATGGTCACACCCTCACCCACTTTCCTGAGTTTTTTCTGGTTTATTAACGT
CAGTCTTTACAGTCAGTGCTCATTGACGGTGGTTTTCTCTGGTTGTTTCCTGAACACGT
AGTGCTCTTAAAGCAATGCCCTGAGGAGAATACAATTCTCCAGGGGCATTCTGATT**GG**
CAGGTGAAGCACAGTGCCATGTTCCCAGCACTGATTTGGGAAGTGGCTTGTCACATCC
CACAGTGAACTCAGTCAACTGGAATGCCTAACTCTCTTTCATAAGACCTCCTGCTACAT
TATGTTTCTCCCAGACTGTACTCAGGTCCAAGAACAGAATTTACTAGTCTATCCTTCTCA
AAGTTCATCATTAAATTCAGTCATTCATCCTAGCCTGTCACATCTACCTGGGGTCATTATT
AGCTATCTCTTCTAGTTTCATATATCTAAACTGGTTCCATCGGTTCTATTTTATAATGCAA
GTACGTAATAGGGTCACGGCTAAAGACACAGCCCTGGAAGGTACTGTTTTTCAAAATG
TACTGTCAACTTACATTTCAGGGCCTCCTCCCAAGGAGAACATGAGACCACTTCATATC
TGGAACTCTAGAAACACTGGCTGCAGCAATCAACCTCCTGTGCCAAGGCTGGCCCCAC
TCTTGTAGCATGGACACTGCTAGCTGGGTTCAAATCCCAGCTCCAGCAGTTGCCAGCG
CTGGGTGACCTCGAGCAAGTGACTACTCCTCACTGGATTGTAGGAGAACTAAATGTGA
TAGTCATTATTTACAGTTCACCACACGCTCTTCACATCCGTTACCTCCCTGGATCCTCAA
AATAACCTCATAATATTGGAAGGCCAACGGACTACTGTCTTTATTTTACAGACAGGGAA
GCTGTGTCTTGTTCTTGGTACCCTGGCACCCCCATGGGAGATTCGGGCTCAGGGGATCC
CCACGCCCGGCGGTCC**CGCACCTGCA**GGGGCACCCCTACCCCCGGCCGCCCCGTACC
TGCAAACACTTGTACAGCACAAAAGCCGCGACGGCCGCGGTGTACAGCGCCCCCAGA

GGCAGCGACCCGGCCCGGGTCCGCTCGCGTTGATCCTTGAGCGGTGGTCGCCGCCGG
GGTTCCGTGGCTCCAGAGCCGGGGTGGGCTGCGTTCCCTGCGGAGGGAGGATACAGG
CGAGTCAGCCGAGCTGGGGCCTGGGACCCGTGCGCAGTCCCAGCCCTCCTCCAGCAG
CCCTAGCTGGTACCTGGGTGTGCCCGGAGGTCGGGATTGGCGCGGTCTCCTAGGACCC
CGGACAGCGCAGACACAAGCAGCAGCCCCACCACACCCAAGAGCGAAACTGCGCCC
GCCATTGCCGGCCCACGGGTGGAGGGATCGGGCGGGCGGTGCCGAAGCGGTCCGGCA
TTGGCCGGCCGCCCCAACGCGCACGCGCACGCGAGCAGGCCGGCCGGCTCCGGGGAG
GCCACGCCCACTCCCCGTAGGGCGGGGCCAGACCATATTTGCATAAGATAGTGTCATTC
TAGCTTTCCTGTATTTGTTCATTTCGTGTCTATTAGCTATTCTGCTAGCCACAATGCCTCT
GAAAGCCTATAGTCTTAGAAAGTTATGCCCGAAAACGGTTTTTTTAATCTCACGCCACC
AACTTTCTCACCCTAATCATAAAACACAATTTCTTTAGGGCTATAAAATACTACTCTGTG
AAGCTGAGGACGTG
