# Supplementary material for: A positive feedback loop of lncRNA-RMRP/ZNRF3 axis and Wnt/β-catenin signaling regulates the progression and temozolomide resistance in glioma
Source: Cell Death Dis. 2021 Oct 16;12(11):952. doi: 10.1038/s41419-021-04245-y (PMC8520527; doi:10.1038/s41419-021-04245-y)
Supplement: Supplementary file 2 — WB data [file 41419_2021_4245_MOESM2_ESM.pptx]

## Slide 1
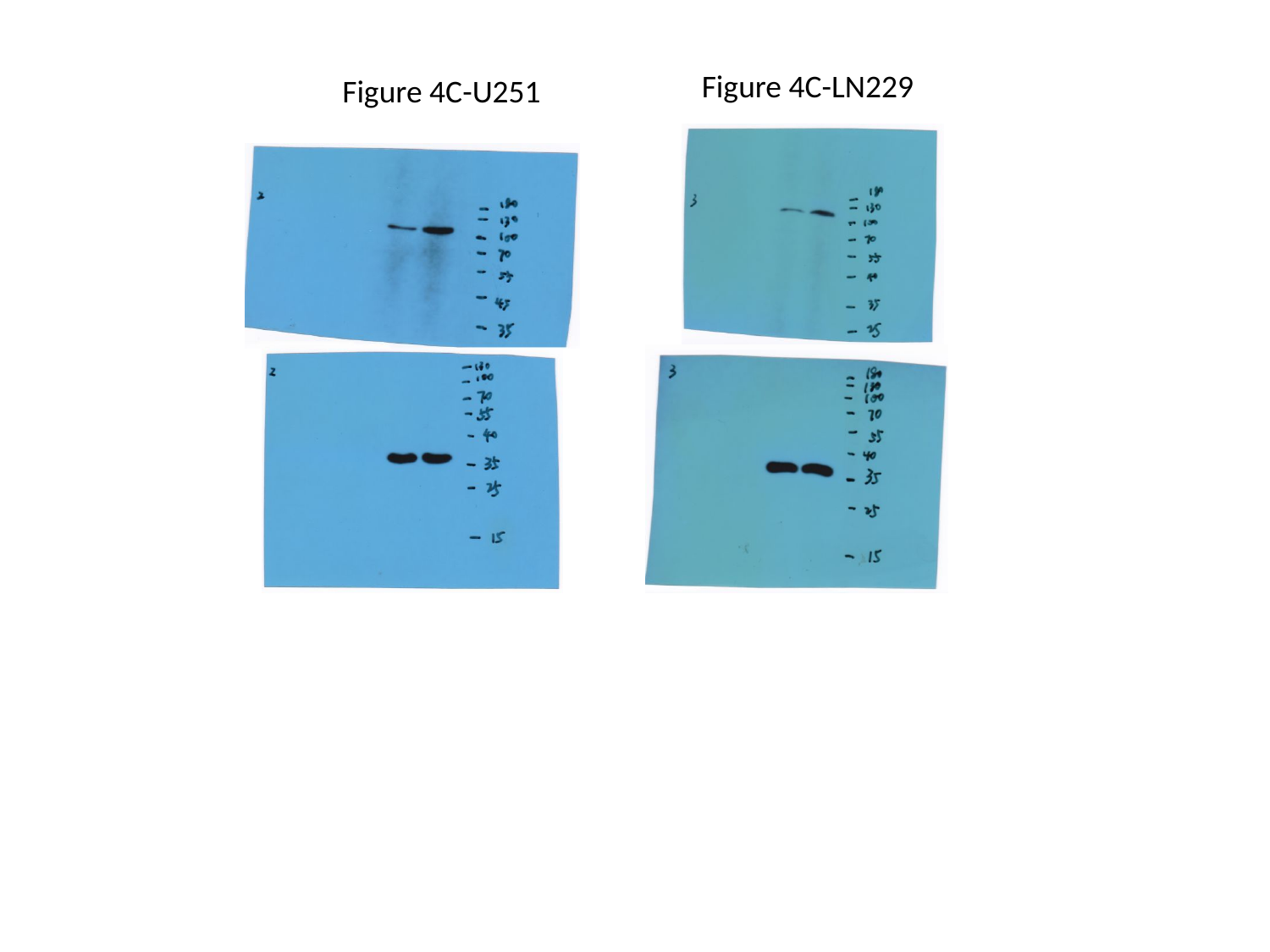

Figure 4C-LN229
Figure 4C-U251

## Slide 2
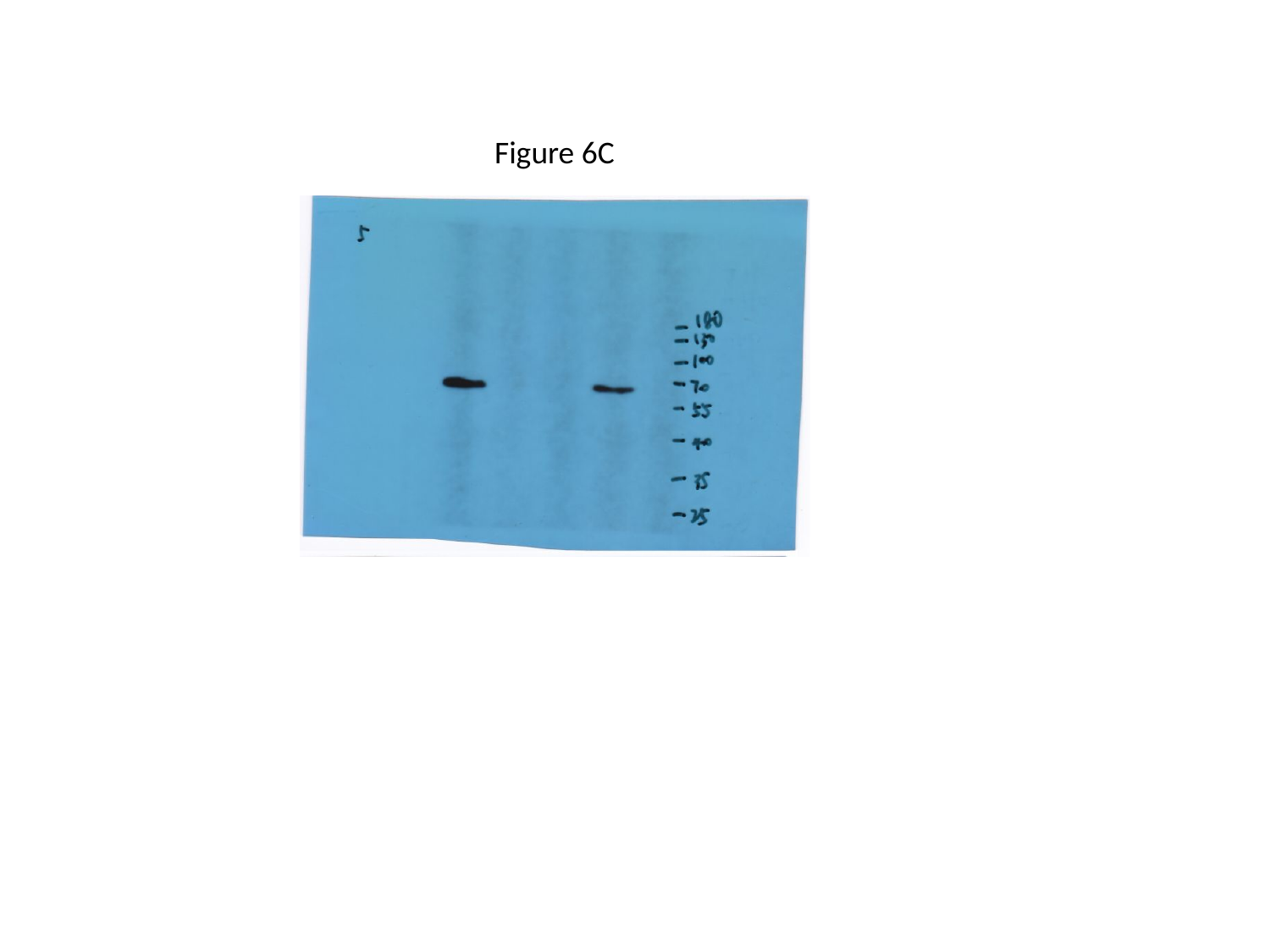

Figure 6C

## Slide 3
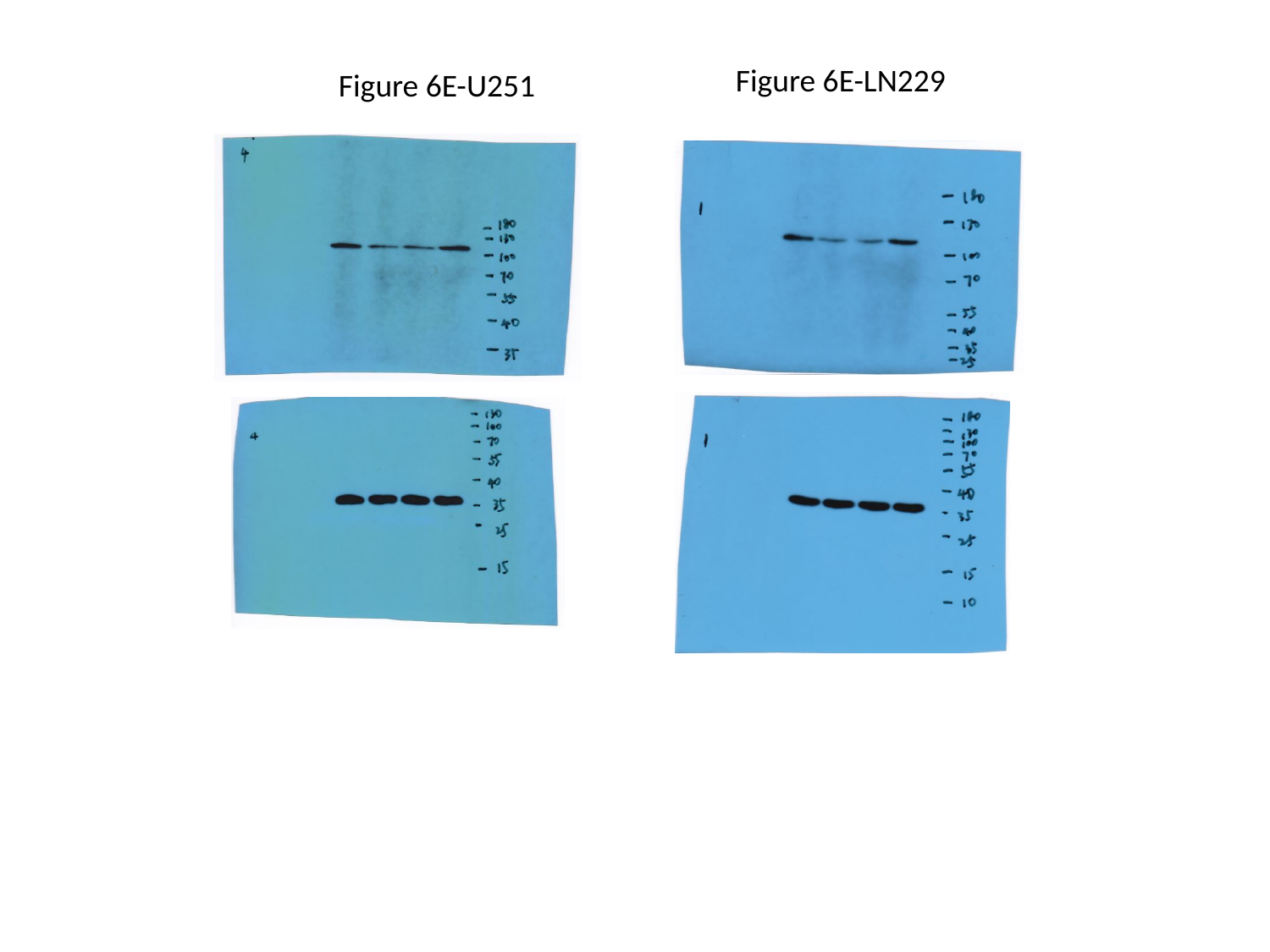

Figure 6E-LN229
Figure 6E-U251

## Slide 4
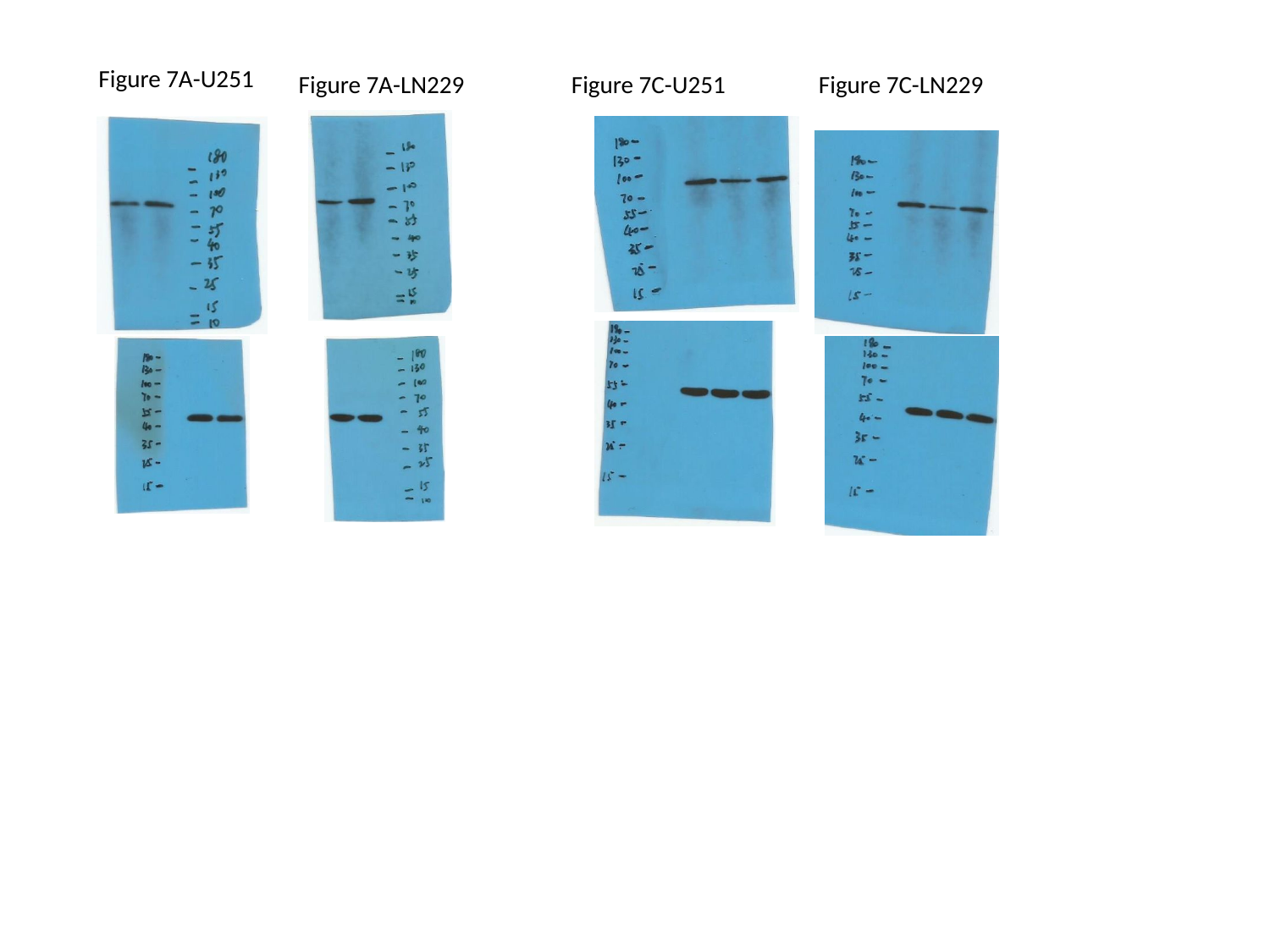

Figure 7A-U251
Figure 7A-LN229
Figure 7C-U251
Figure 7C-LN229
